# Exploring the Effects of Action Observation Therapy on Swallowing Disorders in Stroke: A Functional Connectivity–Based fMRI Study

**DOI:** 10.1155/np/8176431

**Published:** 2025-03-31

**Authors:** Xuting Chen, Xiaolin Sun, Fang Shen, Zhongli Wang, Meihong Zhu, Jianming Fu, Yunhai Yao, Jie Wang, Linhua Tao, Lianjie Ma, Ming Zeng, Xudong Gu

**Affiliations:** Department of Rehabilitation Medicine, The Second Hospital of Jiaxing City, The Second Affiliated Hospital of Jiaxing University, Jiaxing 314000, Zhejiang Province, China

**Keywords:** action observation therapy, degree centrality (DC), dysphagia, functional connectivity (FC), regional homogeneity (ReHo), resting-state functional magnetic resonance imaging, stroke

## Abstract

**Objective:** This study aims to investigate the impact of action observation therapy (AOT) on swallowing disorders following a stroke. Utilizing functional magnetic resonance imaging (fMRI) technology, the study will examine adjustments in brain activity and functional connectivity (FC), providing novel insights for the rehabilitation of swallowing function in stroke patients.

**Methods:** In this study, 11 healthy controls (HCs) and 11 stroke patients were included. The stroke patients underwent a 4-week AOT. To assess the differences in brain region activity between the patients before and after treatment and the HCs, regional homogeneity (ReHo), and degree centrality (DC) were calculated based on fMRI data separately. Important brain regions were selected as regions of interest (ROIs) for subsequent FC analysis, and finally, comparisons were made to evaluate the therapeutic effects.

**Results:** Comparing stroke patients before treatment with HCs, the ReHo values were relatively higher in the inferior temporal gyrus, median cingulate, and paracingulate gyri, and relatively lower in the calcarine fissure and surrounding cortex, middle occipital gyrus, and paracentral lobule. The DC values were relatively higher in the cerebellum, middle frontal gyrus, inferior temporal gyrus, inferior frontal gyrus, orbital part, middle frontal gyrus, and supramarginal gyrus, and relatively lower in the cuneus and paracentral lobule. The FC between the parahippocampal gyrus and the superior parietal gyrus was relatively high, and the FC between the superior occipital gyrus and the superior parietal gyrus was relatively low. Comparing stroke patients after treatment with HCs, the ReHo values were relatively higher in the caudate nucleus, and relatively lower in the cerebellum, superior frontal gyrus, medial orbital, calcarine fissure and surrounding cortex, and middle temporal gyrus. The DC values were relatively higher in the middle frontal gyrus and superior frontal gyrus, and relatively lower in the temporal pole: superior temporal gyrus, calcarine fissure, and surrounding cortex. The FC between the caudate nucleus and the superior parietal gyrus was relatively high, and the FC between the calcarine fissure and surrounding cortex, middle frontal gyrus, orbital part, and the superior parietal gyrus was relatively low. There was no significant difference in ReHo values between stroke patients before and after treatment. The DC value in the superior parietal gyrus increased, and the FC in the superior parietal gyrus and precuneus gyrus was also significantly enhanced before and after treatment.

**Conclusion:** The results of this study indicate that the AOT has a positive effect on enhancing the functional connection and information transmission capabilities of specific brain regions. The impact of this therapy on brain function helps us understand the potential mechanisms of swallowing function network reorganization deeper.

**Trial Registration:** Chinese Clinical Trial Registry: ChiCTR1900021849

## 1. Introduction

Stroke, as a severe neurological disorder, exhibits a high incidence and disability rate worldwide. According to the survey data from the World Health Organization [1], the number of stroke patients worldwide has exceeded 100 million, with over 10 million new cases reported annually. In China, the mortality rate remains alarmingly high, imposing a significant burden on both patients' families and society at large. Following a stroke, individuals not only confront the risk of death but may also experience varying degrees of disability, including motor impairments, dysphagia (DYS), cognitive deficits, and emotional disturbances [2–5]. DYS is one of the prevalent complications; statistics indicate [6] that approximately 50% of stroke survivors continue to experience this condition within 1 week after disease onset. DYS—commonly referred to as swallowing difficulty—entails challenges in safely transporting food or liquids from the mouth to the stomach during ingestion. This condition can arise from multiple factors such as neurological disorders [7, 8], structural abnormalities [9], and muscular dysfunctions. The presence of poststroke DYS adversely impacts patients' nutritional intake and quality of life while increasing their susceptibility to aspiration pneumonia, thereby, presenting significant obstacles to their recovery process.

In clinical practice, common treatment approaches for swallowing disorders include dietary modifications, functional training, and medication therapies [10–12]. However, as swallowing disorders often involve changes in neurocontrol mechanisms, the rapid recovery of swallowing function relies on more specialized or complex treatment methods. Given the severity and prevalence of swallowing disorders following stroke, exploring effective treatment approaches is crucial. Notably, action observation therapy (AOT) [13] has emerged as a valuable intervention in the rehabilitation of swallowing disorders. AOT involves the observation and imitation of specific actions or action sequences to support motor learning and motor skill recovery. In the treatment of swallowing disorders, AOT can facilitate the restoration and improvement of swallowing function by observing and imitating the normal swallowing process. Researchers such as Syrov et al. [14] utilized a P300-based brain–computer interface (BCI) as a feedback system to study the activation of the mirror neuron system (MNS) during the observation of virtual hand movements. They confirmed the positive role of AOT in the recovery of motor function and found that BCI feedback enhanced the effects of passive action observation. Shamili et al. [15] further extended traditional AOT by developing a novel approach called Self-AOT, which emphasized active patient engagement and self-observation. This method allowed patients to better comprehend and perceive their own movement process, enhancing autonomy and self-regulation abilities, and consequently improving rehabilitation outcomes.

Resting-state functional magnetic resonance imaging (rs-fMRI) [16] is a technique employed to elucidate the functional connections and networks within the brain by measuring fluctuations in blood oxygen levels during resting states. This method has gained widespread application in the medical field, providing comprehensive whole-brain functional connectivity (FC) information without necessitating specific tasks, while also facilitating an in-depth analysis of signal consistency across localized brain regions. Regional homogeneity (ReHo) [17] serves as a prevalent regional analytical approach; it quantifies the temporal coherence between each voxel and its neighboring voxels, thereby, revealing synchronous activity within local brain areas. This analytical framework holds significant value for investigating various neurological and psychiatric disorders. Li et al. [18] conducted a comparative study on ReHo and fractional amplitude of low-frequency fluctuations (fALFF) among 22 patients with poststroke DYS versus 30 non-DYS controls. The findings indicated that ReHo values were significantly diminished in the left thalamus, left parietal lobe, and right temporal lobe of the DYS group compared to their non-DYS counterparts; similarly, fALFF values were markedly reduced in both the right middle temporal gyrus and inferior parietal lobule. These results imply that alterations in specific brain regions are closely linked to the severity of swallowing impairments. Degree centrality (DC) [19], an evaluative measure of connectivity, assesses the significance of particular brain regions within the overarching neural network. Jiang et al. [20] contrasted patients with pontine ischemic stroke (PS) and coronary radiation ischemic stroke (CRS) against healthy controls (HCs) using rs-fMRI; they discovered a notable increase in DC within bilateral cingulate gyri and anterior gyri alongside significant decreases observed bilaterally in precuneus areas, calcification zones, and anterior cingulate gyri. Such findings suggest that ischemic strokes profoundly affect FC as well as information transmission across these cerebral territories—potentially correlating with cognitive and perceptual changes following stroke events. FC [21] evaluates synergistic activities among disparate brain regions, illuminating their communication dynamics during resting states. Dai et al.'s [22] exploration into the mechanisms of swallowing dysfunction during the subacute phase of infratentorial strokes revealed enhanced global brain–medulla functional connections, particularly in the precuneus, precentral gyri, and supplementary motor cortices. These enhancements were associated with improved swallowing performance outcomes. This underscores the pivotal role of corticobulbar junctions in the recovery of swallowing functions and presents potential therapeutic targets for intervention strategies.

Thus far, rs-fMRI has been extensively utilized to assess alterations in cerebral function among individuals experiencing swallowing disorders poststroke episodes; research indicates that metrics such as ReHo, DC, and FC can effectively delineate functional impairments across specific neural substrates along with corresponding shifts throughout broader network connectivity—a finding critical for comprehending underlying neural mechanisms governing swallowing difficulties. However, reliance on a single indicator may result in an incomplete understanding of the dynamics within complex brain networks, thereby, potentially failing to uncover all underlying changes in neural function. Considering this, the present study seeks to conduct a comprehensive evaluation of alterations in cerebral functionality among poststroke patients with DYS, employing reference indicators such as ReHo, DC, and FC through both regional and connectivity analyses within the framework of AOT. This approach aims to inform effective treatment strategies for clinical practice.

## 2. Materials and Methods

### 2.1. Participants

**Figure 1:**
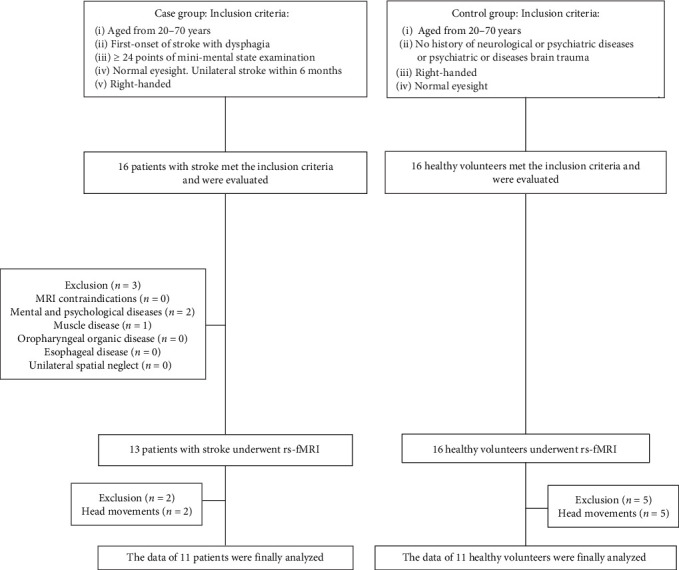
A schematic illustration of the participant selection process used in the present study.

In this study, a total of 32 patients were collected from the Rehabilitation Medical Center of the Second Hospital of Jiaxing from June 2019 to January 2022, and a case–control study was conducted, including 16 HCs and 16 stroke patients. The inclusion criteria of stroke patients meet the latest diagnostic criteria of stroke [23], and all patients with DYS are diagnosed by swallowing radiography. Exclusion criteria: (1) those who can not have magnetic resonance examination. (2) The patient has mental and psychological diseases in the past. (3) At present, the patient has an organic lesion in the oropharynx and cannot cooperate with the examiner. (4) Suffering from esophageal diseases such as gastroesophageal reflux disease. (5) Suffering from muscle diseases such as muscular atrophy in the past. (6) Unilateral spatial neglect inspection cannot be completed. Among the 16 stroke patients, two were excluded due to preexisting mental or psychological disorders, one was excluded because of muscular diseases, and two were excluded owing to excessive head movement during the examination. Additionally, five out of the 16 HCs were excluded for similar reasons related to excessive head movement during their assessment. Consequently, a total of 11 subjects from each group were included in this study. All patients in the group should complete the general condition and swallowing function assessment, including Barthel index [24], nutrition risk screening in 2002 (NRS2002) [25], and Johns Hopkins Fall Risk Assessment Tool (JHFRAT) [26], The Barthel index is utilized to assess the capacity for activities of daily living; a higher score indicates enhanced self-care ability in patients. The NRS2002 score serves as a nutritional risk screening tool, evaluating nutritional status; a higher score signifies an increased nutritional risk for the patient. The JHFRAT score assesses fall risk, where a higher score correlates with an elevated likelihood of falls. Swallowing function was assessed using the Eating Assessment Tool-10 (EAT-10) [27]. This validated rating scale comprises 10 specific items related to DYS, with the patient's swallowing ability evaluated based on their responses. A higher score correlates with a more severe presentation of DYS. This experiment was carried out in full accordance with the moral principles outlined in the Helsinki Declaration. This study received approval from the Ethics Committee of the Second Affiliated Hospital of Jiaxing University (approval number: jxey-2018SKZ03). Each participant signed a formal informed consent form.  Figure 1 depicts the flow chart of the study design.

### 2.2. AOT

First, record a 7-minute video of swallowing action for patients to imitate and observe swallowing action. The contents of the video include: (1) solid food swallowing: observing the model's chewing action and swallowing rice from the front. Then, directly observe the model's chewing movements and swallowing apples. Finally, the model's chewing posture and apple swallowing were observed from the side. (2) Fluid food swallowing: directly observe the posture of the model swallowing yogurt. (3) Empty swallowing: observe the model swallowing three braces directly without drinking water. In all the above videos, the patients in the group should carefully observe the swallowing movements of the model's mouth and throat, the movements of facial muscles, gill muscles, neck muscles and lip muscles, and they should make “E, U, O” sounds with the model. And the stretching movement of the model's front and back tongues. (This experiment refers to the experimental scheme of Zeng Ming, the project research group.)

The single treatment time of this action therapy is 10 min, once a day, 5 days a week for 4 weeks. In addition, all patients in the group received routine swallowing dysfunction training, including sensory stimulation, swallowing posture placement, et cetera, about 30 min, once a day, 5 days a week for 4 weeks.

### 2.3. MRI Data Acquisition

MRI adopts 3.0T equipment (PhilipsNetherlands). Except for the HCs, all patients need to complete MRI examination twice (before and after treatment). All patients in the group need to close their eyes, stay awake, reduce head rotation, and thinking.

The head magnetic resonance examination was performed using a 32-channel head coil from Philips, Netherlands. The scan sequence included the original localization image, high-resolution T1-weighted structural image segmentation, and registration. T1-weighted structural MRI are as follows: sagittal 170 slices, slice thickness 1.0 mm, slice gap 0 mm, repetition time (*T*_R_)/echo time (*T*_E_) = 7.9/3.5 ms, flip angle = 8°, field of view (FOV) = 256 × 256 mm^2^, and matrix = 256 × 256. The following parameters were used to perform conventional T2-weighted imaging fast spin echo sequence to verify the location of the lesion: axial transverse position of 24 slices, slice thickness of 5 mm, gap of 6 mm, *T*_R_ = 3000 ms, *T*_E_ = 80 ms, turnover angle of 90°, FOV = 230 × 190 mm^2^, acquisition matrix of 328 × 224, voxel size of 0.4 × 0.4 × 6 mm^3^, and volume of 3.0. Resting fMRI is obtained by using echo plane imaging sequence: *T*_R_/*T*_E_ = 2000/20 ms, flip angle = 90°, FOV = 240 × 240 mm^2^, matrix = 96 × 96, slice = 46, slice thickness = 2.5 mm, gap = 0.5 mm, and voxel dimensions = 2.5 × 2.5 × 3 mm^3^.

### 2.4. fMRI Data Processing

Statistical analysis Mapping 12 (SPM12) and REST Plus version 1.27 were used to process all functional imaging data.

#### 2.4.1. Data Preprocessing

ReHo and DC: We unified the MRI images of all the patients and removed the first 10 time points. Time-layer correction (time-layer is 46 and scanning sequence is interlayer scanning), participants with maximum translation exceeding 3 mm or rotation exceeding 3° were excluded. All the aligned functional images were normalized in the space of the Montreal Neurological Institute (MNI), the normalized voxel size was 3 × 3 × 3 mm^3^. Removing linear trend of the time course in order to reduce the influence of MRI equipment and regress out the sign, including Friston-24 motion parameters, cerebrospinal fluid (CSF) signals, and white matter (WM). Bandpass temporal filtering (0.01–0.08 Hz) to remove low-frequency drifts and physiological high-frequency noise.

FC: We unified the MRI images of all the patients and removed the first 10 time points. Time-layer correction (time-layer is 46 and scanning sequence is interlayer scanning), participants with maximum translation exceeding 3 mm or rotation exceeding 3° were excluded. Spatial normalization: Using a new segment of the T1 structural image. Alignment of the structural image with the average functional image generates co-receptor T1, which is then divided into six tissues: gray matter, WM, CSF, meninges, skull, and extracerebral cortex. Finally, the probability map of the divided tissues is written into the functional image. Spatial smoothing: spatial smoothing with an isotropic Gaussian kernel with a full width at half maximum (FWHM) of 6 mm × 6 mm × 6 mm. Removing linear trend of the time course in order to reduce the influence of MRI equipment and regress out the sign, including Friston-24 motion parameters, CSF signals, and WM. Bandpass temporal filtering (0.01–0.08 Hz) to remove low-frequency drifts and physiological high-frequency noise.

#### 2.4.2. Indicator Calculation

The ReHo index of 27 voxel connections was calculated, and each subject got a ReHo map. mReHo is obtained by dividing the original ReHo value of each voxel by the average ReHo value of all voxels in the brain. Subsequently, the mReHo image is smoothed with a 6 mm smoothing kernel and the mReHo diagram was obtained for each subject. The mReHo diagrams were used for subsequent statistical analysis.

The DC index is calculated for the filtered (0.01–0.08 Hz) data using the DC intensity value (DC weighted), *r* = 0.25. Each subject gets a DC map, DC averaging, get each subject's mDC map, then, use 6 mm smoothing check mDC map for smoothing, subsequent master plan analysis using mDC map.

Region of interest (ROI) selection: The brain regions with the difference of voxel *p*  < 0.01 and cluster *p*  < 0.05 before and after DC treatment were made into masks, as the ROI of functional connection. After filtered wave (0.01–0.08 Hz) data, using ROI as the seed point, and brain-wide FC indicator calculation, each subject obtains a FC diagram, and Fisher Z transformation of FC obtains each subject's zFC diagram, and subsequent statistical analysis uses the zFC diagram. (Note: Since only DC was significantly different before and after treatment in pretreatment, only the intergroup differences in DC brain regions were used for ROI.)

### 2.5. Statistical Analysis and Multiple Comparison Corrections

#### 2.5.1. Statistical Analysis

Using SPSS 26.0 version of statistical software for data analysis, all measurement data were calculated using the mean ± standard deviation. Paired *T*-test was used for the data that followed the normal distribution before and after treatment, and rank sum test was used for the data that did not conform to the normal distribution. The comparison between the two groups of patients satisfied the normal distribution and homogeneity of variance, and the independent sample *T*-test was used. *p*  < 0.05 was considered statistically significant.

Statistical analysis of ReHo:

1. Two-sample *T*-test was performed on mReHo images of patients before treatment and control group and the lesion images of patients were regressed as covariate.
2. Make paired sample *T*-test on the mReHo images of the patient group after treatment and before treatment.
3. Make two-sample *T*-test on the mReHo images of the patient group after treatment and the control group and use the patient's lesion image as a covariate for regression.

DC statistical analysis:

1. Two-sample *T*-test on SmDC-weighted images of patients before and after treatment and control group and use the patient's lesion map as a covariate for regression.
2. Paired sample *T*-test on SmDC-Weighted images of patients after treatment and control group.
3. Two-sample *T*-test on SmDC-Weighted images of patients after treatment and control group and use the patient's lesion map as a covariate for regression.

FC statistical analysis:

1. Two-sample *T*-test was performed on the zFC images of the patients before treatment and the control group and the patient's lesion image was used as a covariate for regression.
2. Paired sample *T*-test was performed on the zFC images of the patients after treatment and before treatment.
3. Two-sample *T*-test was performed on the zFC images of the patient group and the control group after treatment and the patient's lesion images were regressed as covariates.

#### 2.5.2. Multiple Comparison Corrections

ReHo multiple comparison correction: GRF correction was used for the results of two-sample *T*-test, with voxel *p*  < 0.01 and cluster *p*  < 0.05, to see if there were any significant difference lumps left.

DC multiple comparison correction: GRF correction was used for the results of two-sample *T*-test, with voxel *p*  < 0.01 and cluster *p*  < 0.05, to observe whether there were significant difference lumps left.

FC multiple comparison correction: GRF correction was used for the results of two-sample *T*-test, with voxel *p*  < 0.01 and cluster *p*  < 0.05, to observe whether there were any significant difference lumps left.

## 3. Results

### 3.1. Demographic and Clinical Characteristics

The demographics of the subjects and the treatment effect are compared between Tables 1 and 2. Prior to treatment, there was no significant difference in sex ratio between patients and HCs; however, notable disparities were observed in age, education years, Barthel index, NRS2002 score, JHFRAT, and EAT-10 score. With respect to alterations in swallowing function, the results indicated that for the stroke patients before treatment the EAT-10 score was 31.55 ± 4.37 points, while it decreased significantly to 20.27 ± 4.54 points after treatment. The difference was statistically significant (*p*  < 0.05), indicating a substantial improvement in swallowing function.

### 3.2. Results of ReHo Analysis

**Figure 2:**
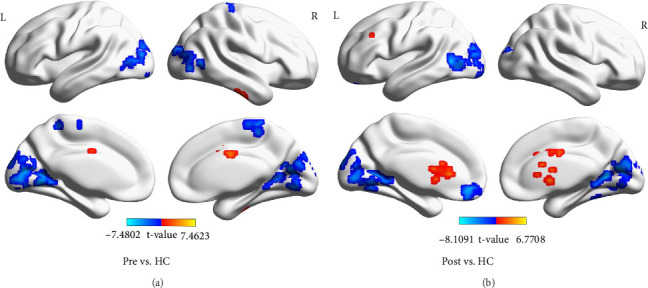
Brain region differences of regional homogeneity (ReHo) values between stroke patients before treatment, stroke patients after treatment, and healthy controls (HCs). (A) The differences of ReHo values between stroke patients before treatment and HCs. (B) The differences of ReHo values between stroke patients after treatment and HCs (GRF, voxel level *p*  < 0.01, and cluster level *p*  < 0.05).

Compared to HCs, stroke patients before treatment exhibited increased ReHo values in the inferior temporal gyrus and paracingulate gyrus of medial nucleus. Conversely, decreased ReHo values were observed in the calcarine fissure and surrounding cortex, middle occipital gyrus, and paracentral lobule (Table 3 and Figure 2A); stroke patients after treatment showed increased ReHo values in the caudate nucleus compared to HCs. However, decreased ReHo values were found in the cerebellum, superior frontal gyrus, medial orbital, calcarine fissure and surrounding cortex, and middle temporal gyrus. Notably, no significant differences were detected before and after treatment based on ReHo statistics (Table 3 and Figure 2B).

### 3.3. Results of DC Analysis

**Figure 3:**
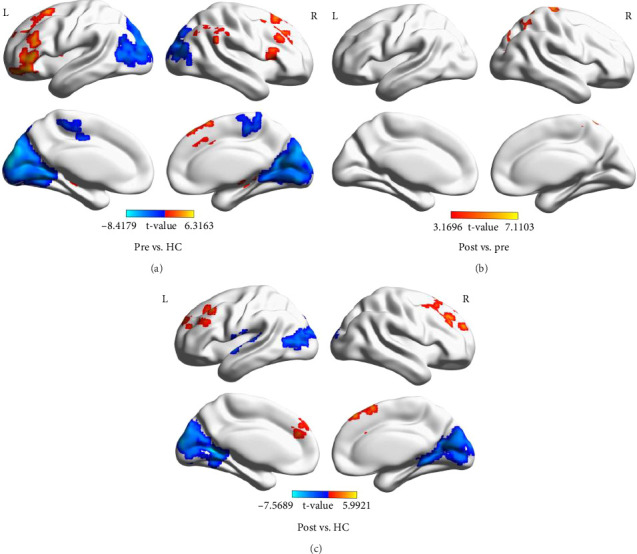
Brain region differences of degree centrality (DC) values between stroke patients before treatment, stroke patients after treatment, and healthy controls (HCs). (A) The differences of DC values between stroke patients before treatment and HCs. (B) The differences of DC values between stroke patients before treatment and after treatment. (C) The differences of DC values between stroke patients after treatment and HCs (GRF, voxel level *p*  < 0.01, and cluster level *p*  < 0.05).

Compared to HCs, stroke patients before treatment exhibited increased DC values in the cerebellum, middle frontal gyrus, inferior temporal gyrus, inferior frontal gyrus, orbital part, middle frontal gyrus, and supramarginal gyrus. Conversely, decreased DC values were observed in the cuneus and paracentral lobule; stroke patients after treatment showed increased DC values in the middle frontal gyrus and superior frontal gyrus compared to HCs. However, decreased DC values were found in the temporal pole: middle temporal gyrus, calcarine fissure, and surrounding cortex. In DC statistics, we found that the superior parietal gyrus showed significant differences before and after treatment (Table 4 and Figure 3).

### 3.4. Results of FC Analysis

**Figure 4:**
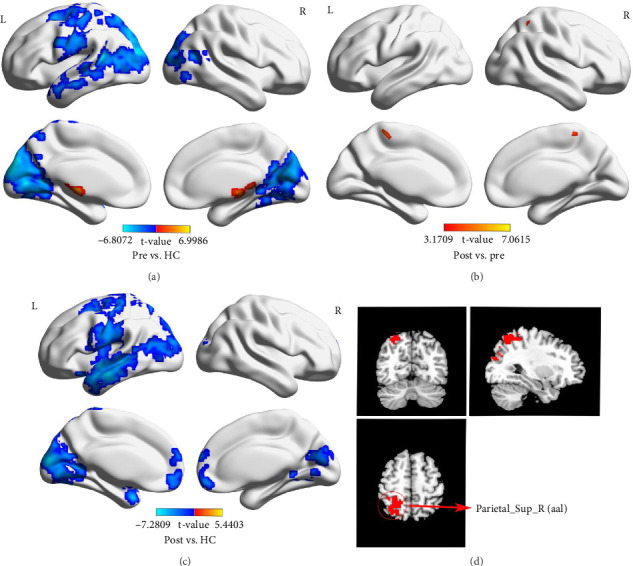
Brain region differences of functional connectivity (FC) values between stroke patients before treatment, stroke patients after treatment, and healthy controls (HCs). (A) The differences of FC values between stroke patients before treatment and HCs. (B) The differences of FC values between stroke patients before treatment and after treatment. (C) The differences of FC values between stroke patients after treatment and HCs. (D) Region of interest (ROI; GRF, voxel level *p*  < 0.01, and cluster level *p*  < 0.05).

We choose the superior parietal gyrus (33, −66, 57; Table 4 and Figure 4) as the ROI for analyzing whole-brain FC. Compared to HCs, FC was significantly increased in the superior parietal gyrus and parahippocampal gyrus in stroke patients before treatment, while FC was significantly decreased in the superior parietal gyrus and superior occipital gyrus. Compared to HCs, FC was enhanced in the superior parietal gyrus and caudate nucleus in stroke patients after treatment, while FC was decreased in the calcarine fissure and surrounding cortex, middle frontal gyrus, orbital part, and superior parietal gyrus. In addition, FC in the superior parietal gyrus and precuneus gyrus was also significantly enhanced before and after treatment (Table 5 and Figure 4).

## 4. Discussion

The results of this study showed that the swallowing function of patients with swallowing disorder after stroke was significantly improved after a period of intervention with AOT. Meanwhile, we also observed that the FC of some brain regions was significantly enhanced before and after treatment by using rs-fMRI. It mainly includes parietal gyrus, precuneus, caudate nucleus, middle frontal gyrus, and superior frontal gyrus. We believe that this may be associated with improved swallowing function after stroke.

In the comparison between the two groups of patients before and after treatment, we found that the DC of the superior parietal gyrus was directly enhanced with statistical significance, so we speculated that the superior parietal gyrus played an important role in the functional recovery in this study. From the current understanding of brain regions, the parietal gyrus plays an important role in motor control. It is recognized as one of the pivotal areas involved in the analysis of sensory information [28]. The study of Buneo and Andersen [29] revealed the role of superior parietal gyrus in arm movement planning. The superior parietal gyrus is a critical region involved in motor control, particularly known for its role in coordinate transformation, and is considered one of the key areas in the brain for these functions. They found that the superior parietal gyrus is capable of transforming information from diverse coordinate systems, enabling interaction between different reference frames. In arm motion, the superior parietal gyrus can transfer the target position in the eye-centered frame of reference to the hand-centered frame of reference for processing. The superior parietal gyrus is demonstrated to integrate visual information with motor commands, thereby, maintaining an accurate assessment of the arm's position and dynamically refining the trajectory plan. Therefore, it is speculated that during the treatment of motion observation therapy in this study, the parietal gyrus plays a crucial role in processing sensory information, which helps to transform the observed swallowing information into specific motor behaviors, such as laryngeal elevation, oral transport, et cetera [30]. In addition, the improvement of DC value also indicates that the connection ability between the superior parietal gyrus and some other motor cortex is enhanced. It acts as a bridge throughout the process, combining sensation and movement to achieve enhanced control over the ability to swallow. Another point of concern is that in our study, we also observed changes in the precuneus gyrus, which is usually relatively easy to be ignored because of its special location, but Cavanna and Trimble [31] pointed out that on the one hand, the precuneus gyrus plays an important role in highly integrated tasks and the recovery of patients with DYS after stroke also requires complex coordination and regulation. By enhancing the transmission of information with other brain regions, the precuneus gyrus may coordinate the motor planning, sensory feedback, and execution processes related to swallowing, thus, improving the swallowing function of patients with DYS after stroke. On the other hand, the precuneus gyrus also plays an important role in self-awareness and sensory experience, which may help patients to participate and perceive more actively when swallowing.

Some of the brain regions mentioned above may be more involved in or regulate swallowing itself. On the other hand, some brain regions may indirectly regulate swallowing function in other ways. The middle frontal gyrus [32] is recognized as being involved in advanced cognitive processes and is crucial for the seamless completion of the swallowing sequence. The improvement of cognitive ability can have a clearer cognitive level when identifying food masses or performing swallowing movements and achieve the effect of improving swallowing function. The caudate nucleus, as part of the basal ganglia system, is generally considered to be associated with airway protection during swallowing. Damage to the caudate nucleus may make it easier for food or liquid to seep into the airway, increasing the risk of aspiration. Research [33] indicates that in clinical settings, the functional status of the caudate nucleus should be considered in assessments of swallowing function. Furthermore, targeted interventions may be necessary to enhance airway protection during swallowing. Therefore, we can speculate that the increase of ReHo value of caudate nucleus may be an indirect improvement of swallowing function by reducing the risk of aspiration.

Additionally, AOT is a form of rehabilitation treatment that seeks to foster motor recovery and functional enhancement in patients by having them observe others engaged in exercises. Rooted in the theory of the MNS, this approach posits that when individuals observe movements, their brain's mirror neurons are activated, generating neural activity patterns similar to those produced during actual physical activity. In the research conducted by Ushioda et al. [34], it was observed that the BA6 and BA40 brain regions are significantly activated prior to the onset of swallowing-related animation stimulation and the subsequent actual throat actions. This finding suggests the presence of MNS activity within these regions. In the process of watching the animation, the MNs is activated to imitate or simulate the observed related movements. This conclusion and our findings support each other, and the enhanced functional connections in areas such as the superior parietal gyrus and the superior frontal gyrus may also benefit from the activation of the MNs. In summary, the theory of AOT and our results support each other, suggesting that the enhancement of functional connections in areas such as the superior parietal gyrus and superior frontal gyrus may be related to the activation of the MNs. These findings also provide a valuable structural reference for the improvement of rehabilitation therapy.

This study does have several limitations that should be acknowledged. First, the sample size employed in this research was small, which could potentially constrain the study's ability to provide robust and widely applicable findings. Second, the absence of a standardized control group in our investigation—that is, a group of stroke patients with DYS who received only traditional forms of treatment—may hinder our comprehensive assessment of the efficacy of AOT from the interpreted results. Last, the lack of long-term follow-up in this study limits our understanding of the enduring effects of AOT on swallowing function. Consequently, it is anticipated that future research will aim to enlarge the sample size, incorporate a control group for comparative analysis and include long-term follow-up to monitor the sustained impact of AOT on patients' swallowing abilities.

## 5. Conclusion

Our previous research [35] has demonstrated that AOT can significantly improve swallowing function in poststroke patients experiencing DYS. The study primarily indicates that this therapeutic approach positively influences the FC among different brain regions, which may be intricately linked to the restoration of swallowing capabilities.

## Figures and Tables

**Table 1:** Clinical characteristics of stroke patients and healthy controls (HCs).

Group	Course of disease (day)	Stroke classification		Lesion site		
		Cerebral infarction	Cerebral hemorrhage	Lateral Ventricle	Frontal/temporal/parietal lobe	Basal ganglia
HCs						
Stroke patients	38.36 ± 17.72^*∗*^	8^*∗*^	3^*∗*^	2^*∗*^	4^*∗*^	5^*∗*^

*Note:* The stroke classification and lesion site between the two groups was compared using the chi-square test, and the course of disease were compared using the independent two-sample *T*-test.

*⁣* ^*∗*^ *p<*0.001, there was a statistical difference between the two groups.

**Table 2:** Demographic characteristics and clinical scores of stroke patients and healthy controls (HCs).

Demographic and clinical parameters	Stroke patients (*n* = 11)	HCs (*n* = 11)	Test statistic	*p* Value
Age (year)	65.45 ± 10.25	52.27 ± 13.04	2.635	0.016
Gender (male/female)	8/3	5/6	1.692	0.193
Edu years (year)	6.09 ± 1.87	11.45 ± 3.75	−4.245	0.001
General function				
Barthel index	65.27 ± 9.95	100	−11.575	<0.001
Nutritional Risk Screening 2002 (NRS2002)	1.82 ± 1.66	0	3.627	0.006
John Hopkins Fall Rish Assessment Tool (JHFRAT)	10.18 ± 4.07	0	8.297	<0.001
Swallowing function				
Eating assessment tool-10 (EAT-10)	Pretreatment 31.55 ± 4.37	0	23.957	<0.001
	Posttreatment 20.27 ± 4.54	0	14.808	<0.001

*Note:* The gender distribution between the two groups was compared using the chi-square test, and the age, education year, Barthel index, NRS2002, JHFRAT, and EAT-10 scores were compared using the independent two-sample *T*-test. A paired *T*-test was used to compare the EAT-10 scores for the stroke group before and after treatment.

**Table 3:** Pairwise comparison of ReHo values between the three groups.

Contrast	Brain regions	Cluster size	MNI coordinates			*T*-value
			*X*	*Y*	*Z*	
Pre-HC	Temporal_Inf_R (aal)	72	57	−15	−39	7.4623
	Calcarine_L (aal)	640	0	−78	9	−7.4802
	Cingulum_Mid_R (aal)	215	24	−15	9	5.3196
	Occipital_Mid_L (aal)	53	−33	−87	12	−5.3028
	Paracentral_Lobule_R (aal)	118	9	−36	54	−4.3304

Post-HC	Cerebelum_6_R (aal)	65	27	−48	−27	−5.3727
	Frontal_Med_Orb_L (aal)	78	−6	48	−9	−8.1091
	Calcarine_L (aal)	471	0	−78	9	−7.7972
	Temporal_Mid_L (aal)	199	−51	−63	6	−6.8093
	Caudate_L (aal)	532	12	3	33	6.7708

*Note: T*, statistical value of ReHo differences between the two groups (negative values: the former < the latter; positive values: the former > the latter); MNI, MNI coordinate system or template; Temporal_Inf (aal), inferior temporal gyrus; Calcarine (aal), calcarine fissure and surrounding cortex; Cingulum_Mid (aal), median cingulate and paracingulate gyri; Occipital_Mid (aal), middle occipital gyrus; Paracentral_Lobule (aal), paracentral lobule; Cerebelum_6 (aal), superior cerebellum; Frontal_Med_Orb (aal), middle frontal gyrus, orbital part; Temporal_Mid (aal), middle temporal gyrus; Caudate_L (aal), caudate nucleus.

Abbreviations: HC, healthy control; MNI, Montreal Neurological Institute; ReHo, regional homogeneity.

**Table 4:** Pairwise comparison of DC values between the three groups.

Contrast	Brain regions	Cluster size	MNI coordinates			*T*-value
			*X*	*Y*	*Z*	
Pre-HC	Cerebelum_Crus2_L (aal)	235	−42	−72	−39	4.5888
	Frontal_Mid_R (aal)	1,157	42	24	54	5.7974
	Temporal_Inf_L (aal)	149	−39	−27	−6	5.4142
	Frontal_Inf_Orb_L (aal)	291	−42	39	−18	6.0705
	Cuneus_R (aal)	3,482	12	−93	15	−8.4179
	Frontal_Mid_L (aal)	355	−24	18	6	6.3163
	SupraMarginal_R (aal)	225	66	−48	30	4.8905
	Paracentral_Lobule_L (aal)	150	0	−27	54	−4.4838

Post-pre	Parietal_Sup_R (aal)	440	30	−66	57	7.1103

Post-HC	Temporal_Pole_Sup_L (aal)	179	−60	6	−3	−5.7976
	Calcarine_L (aal)	1780	−21	−57	6	−7.5689
	Frontal_Mid_L (aal)	292	−36	−18	27	5.9921
	Frontal_Sup_L (aal)	182	−15	51	30	5.0506
	Frontal_Mid_R (aal)	498	33	6	48	5.3835

*Note:* Cerebelum_Crus2_L (aal), inferior cerebellum; Frontal_Mid (aal), middle frontal gyrus; Temporal_Inf (aal), inferior temporal gyrus; Frontal_Inf_Orb (aal), inferior frontal gyrus, orbital part; Cuneus (aal), cuneus; SupraMarginal (aal), supramarginal gyrus; Paracentral_Lobule (aal), paracentral lobule; Parietal_Sup (aal), superior parietal gyrus; Temporal_Pole_Sup (aal), temporal pole: superior temporal gyrus; Calcarine (aal), calcarine fissure and surrounding cortex; Frontal_Sup (aal), superior frontal gyrus.

Abbreviations: DC, degree centrality; HC, healthy control; MNI, Montreal Neurological Institute.

**Table 5:** Pairwise comparison of FC values between the three groups.

Contrast	Brain regions	Cluster size	MNI coordinates			*T*-value
			*X*	*Y*	*Z*	
Pre-HC	ParaHippocampal_R (aal)	158	12	−21	−18	6.9986
	Occipital_Sup_L (aal)	5,014	−15	−87	21	−6.8072

Post-pre	Precuneus_L (aal)	135	−3	−39	63	7.0615

Post-HC	Calcarine_L (aal)	3,525	−6	−90	12	−7.2809
	Frontal_Med_Orb_L (aal)	198	−3	57	−12	−4.2311
	Caudate_R (aal)	134	18	9	24	5.4403

*Note:* ParaHippocampal (aal), parahippocampal gyrus; Occipital_Sup (aal), superior occipital gyrus; Precuneus (aal), precuneus; Calcarine (aal), calcarine fissure and surrounding cortex; Frontal_Med_Orb (aal), middle frontal gyrus, orbital part; Caudate_L (aal), caudate nucleus.

Abbreviations: FC, functional connectivity; HC, healthy control; MNI, Montreal Neurological Institute.

## Data Availability

The corresponding author can provide you with the data that were utilized to support the study's conclusions upon request.
